# The Role of Epstein–Barr Virus in Cervical Cancer: A Brief Update

**DOI:** 10.3389/fonc.2018.00113

**Published:** 2018-04-17

**Authors:** Semir Vranic, Farhan Sachal Cyprian, Saghir Akhtar, Ala-Eddin Al Moustafa

**Affiliations:** College of Medicine, Qatar University, Doha, Qatar

**Keywords:** cervical cancer, virus, human papilloma virus, Epstein–Barr virus, carcinogenesis

## Abstract

Epstein–Barr virus (EBV) belongs to the group of gamma-herpes viruses and was the first recognized human oncovirus. EBV is responsible for infectious mononucleosis and multiple lymphoid and epithelial malignancies including B-cell lymphomas (Burkitt lymphoma, Hodgkin lymphoma, and post-transplant lymphoproliferative disorder), various T-cell/NK lymphoproliferative disorders, nasopharyngeal carcinoma, and gastric carcinoma, respectively. In addition, the presence of EBV has been documented in other cancers including breast, prostate, oral, and salivary gland carcinomas. The presence and role of EBV in cervical cancer and its precursor lesions (CIN) have also been described, but the results from the literature are inconsistent, and the causal role of EBV in cervical cancer pathogenesis has not been established yet. In the present review, we briefly surveyed and critically appraised the current literature on EBV in cervical cancer and its variants (lymphoepithelioma-like carcinoma) as well as its precursor lesions (CIN). In addition, we discussed the possible interactions between EBV and human papilloma virus as well as between EBV and immune checkpoint regulators (PD-L1). Though further studies are needed, the available data suggest a possible causal relationship between EBV and cervical cancer pathogenesis.

## Introduction

Infectious agents contribute to approximately 18% of all cancers worldwide (1). These agents include bacteria (e.g., *Helicobacter pylori*), viruses [human papilloma virus (HPV), hepatitis B virus, hepatitis C virus, Epstein–Barr virus (EBV), human herpes virus-8, human T-cell lymphotropic virus-1 (HTLV-1), and Merkel cell polyomavirus], and parasites (e.g., Schistosoma and liver flukes) (1–7). The cancers associated with the abovementioned infections include several hematologic {lymphomas/lymphoproliferative disorders [Hodgkin lymphoma (HL), Burkitt lymphoma, post-transplant lymphoproliferative disorder, various T-cell/NK lymphoproliferative disorders]} and solid malignancies (carcinomas: nasopharyngeal, hepatocellular, gastric, cervical, Merkel cell, and bladder carcinoma). In addition, the presence of diverse microbial agents (e.g., viruses SV40, BK, JCV, and HTLV-II) has been described in many other cancer subtypes, but the results and causal relationships are inconsistent and inconclusive (7).

Viral infections are the most common cause of infection-related cancer agents (~12–15%) (4, 8). The vast majority of these infections occur in developing countries although the frequency is not negligible in developed world (5, 9). HPVs along with EBV are associated with 38% of all virus-associated cancers (8). Most viral-associated cancers develop after long-term latency (15–40 years) (10). Notably, viral infections within the cancer cells are not mutually exclusive, and synergistic oncogenic effects can and likely occur (see also the paragraph on EBV in cervical cancer) (8, 11). In addition, endemic forms of Burkitt lymphoma (mainly in equatorial Africa), an EBV-associated malignancy, are frequently linked to coinfection with the malaria-causing bacterium called *Plasmodium falciparum* (1).

### EBV and Cancer

**Figure 1:**
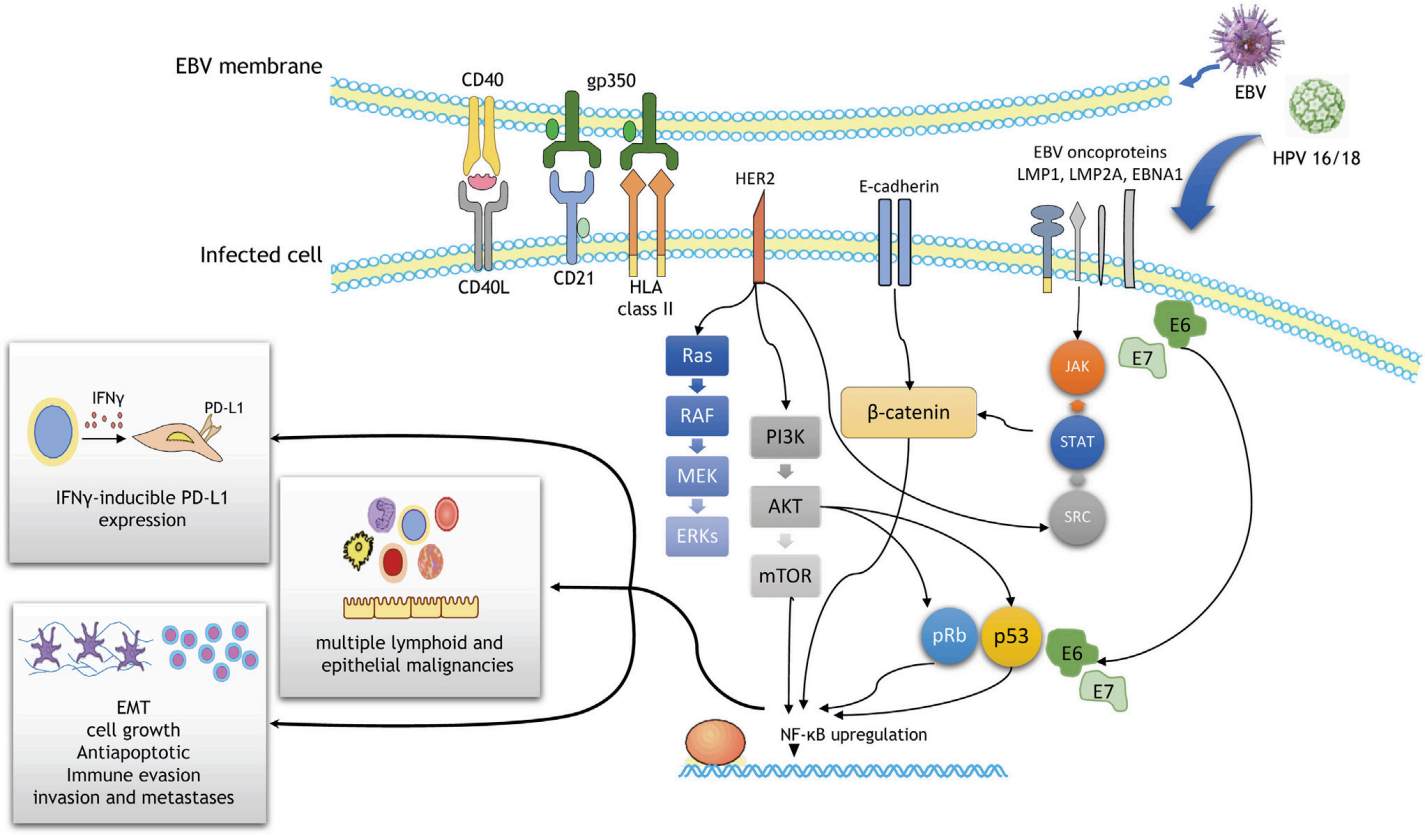
Schematic outline showing a potential cooperation between Epstein–Barr virus (EBV) and human papilloma virus (HPV) oncoproteins to initiate cancer development and enhance malignancy progression. Efficient infection involves the activation of the PI3K/AKT, MAPK/ERK, JAK/STAT, β-catenin, and p53 pathways. As a result, NF-ĸB upregulation induces cell proliferation, epithelial–mesenchymal transition (EMT), and immune evasion through IFNγ-inducible PD-L1 expression.

Epstein–Barr virus, previously known as human herpesvirus-4, is the first recognized human oncovirus. It belongs to the group of gamma-herpes viruses and is ubiquitously present in the adult population *via* salivary transmission (2). Upon infection, EBV typically remains in memory B-cells (Figure 1) in a latent phase, but may also be detected in epithelial cells (oropharynx) as well as in subsets of T-cells and NK-cells (2, 3, 5). EBV causes infectious mononucleosis and multiple lymphoid and epithelial malignancies including B-cell lymphomas (Burkitt lymphoma, HL, and post-transplant lymphoproliferative disorder), various T-cell/NK lymphoproliferative disorders, and nasopharyngeal and gastric carcinomas (1, 12, 13).

The EBV genome is composed of double-stranded DNA, measuring approximately 172 kb in length (2). EBV encodes several viral oncogenes including EBV-encoded nuclear antigens [EBNA (1–3)] and the latent membrane proteins [LMP (1–2)] (14). Interactions of its surface protein gp350 with CD21 receptor and HLA class II on B-lymphocytes represent the major mechanisms of the entrance into B-cells (Figure 1) (2). Upon primary infection and replication, most of its genes are turned off and the virus switches to the latent phase.

Molecular events related to EBV roles in cancer have been well described in HL and undifferentiated variants of nasopharyngeal carcinoma. Approximately 50% of HLs are EBV-positive, particularly lymphocyte-depleted and mixed-cellularity variants of HL. Reed–Sternberg (RS) cells, typical B-transformed neoplastic cells in HL, are infected by EBV. RS activation and survival are largely dependent on NF-ĸB upregulation that is mediated by the interaction of CD40 receptor and LMP1 oncoprotein of EBV (Figure 1) (15). Other signaling pathways may also be activated by this interaction including MAPK/ERK, PIK3CA/AKT, JAK/STAT, and Notch pathways (Figure 1) (2). Apart from LMP1 oncoprotein, EBNA1 is another important EBV product that is required for the replication and maintenance of EBV genome (2). In the case of undifferentiated nasopharyngeal carcinoma, LMP1, LMP2, and EBNA1 products of EBV have been shown to promote cell growth and exert antiapoptotic effects in neoplastic cells (16), while LMPA2A prevents epithelial differentiation of the cells (14). They are also involved in cancer progression, invasion, and metastases as well as immune evasion, all the features that contribute to a highly aggressive behavior of nasopharyngeal carcinoma (16). Several recent studies also highlight novel mechanisms on the complex interplay between viruses (including EBV and HPV), immune system, and carcinogenesis. Among these, apolipoprotein B mRNA editing enzymes (APOBEC) family of deaminases appears to play a prominent role (17, 18). APOBEC family of enzymes, involved in the editing of DNA and/or RNA sequences, acts on the inner immune system against viruses and endogenous retroelements (19). A study of Kalchschmidt et al. showed that EBV (*via* its protein EBNA3C) may upregulate one of the APOBEC enzymes called activation-induced cytidine deaminase in EBV-infected B-lymphocytes (18). This may lead to somatic hypermutations at the IgH locus of B-lymphocytes and consequently induce progression of EBV-infected B-lymphocytes into neoplastic B-cells (lymphomas) (18). Similarly, Chen et al. demonstrated the importance of APOBEC enzymes in mediating the complex interactions between HPV infection, host immune system, and cervix during cervical cancer progression (17).

Epstein–Barr virus expression has also been described in several other solid malignancies including breast, prostate, oral, and salivary gland carcinomas (20–23).

Of note, EBV vaccines aimed to prevent primary infection and to treat EBV-related malignancies have been developed but still not approved (24, 25). The prophylactic vaccines have focused on EBV gp350 protein, which represents the major target of neutralizing antibodies while therapeutic vaccines targeted LMP2 and EBV nuclear antigen-1 (24). Thus, a phase 2 clinical trial conducted by Sokal et al. showed that the EBV vaccine had reduced the rate of infectious mononucleosis, but not the EBV infection (26). On the other hand, the studies have shown that the infusion of EBV-specific T cells may be effective in the treatment of EBV-associated malignancies such as HL and nasopharyngeal carcinoma (24). Given the frequency of EBV-associated cancers, the EBV vaccines are urgently needed.

## Role of EBV in Cervical Cancer: Possible Oncogenic Effects of HPV/EBV Interactions

Cervical cancer is the fourth-most common and fourth-most deadly female malignancy worldwide (27, 28). In developing countries, it is the most common cancer subtype and the third leading cancer-mortality causes (28). The vast majority (more than 95%) of the cervical cancers (squamous cell carcinomas and adenocarcinomas) has been attributed to the infection with high-risk HPVs (14, 27), which are now considered a major cause of cervical cancer (12). Numerous high-risk HPVs have been linked to cervical cancer including HPV types 16, 18, 31, 33, 35, 39, 45, 51, 52, 56, 58, and 59 (these viruses are allocated by IARC in Group 1 given that their carcinogenicity has been sufficiently demonstrated) (7). However, most common (~70%) HPV types involved in cervical carcinogenesis are HPV16 and 18 (29). It is well known that high-risk HPVs act *via* their proteins E6 and E7 that interact with p53 and pRb affecting the cell cycle, apoptosis, and cell adhesion (Figure 1) (11, 29, 30).

Previous data indicate that other infectious agents may also be actively involved in cervical carcinogenesis (12). Among these, EBV appears to be one of the most relevant. Some, but not all early studies, published more than two decades ago, offered the evidence of EBV DNA presence in both precancerous (CIN) and invasive cervical carcinoma cells (31–34), suggesting its possible role in the pathogenesis of cervical carcinoma. Other studies revealed the presence of EBV in inflammatory, but not in cervical cancer cells (35–39).

A recently published meta-analysis (12), based on 25 publications, revealed the pooled prevalence of EBV in cervical cancer to be 43.63%, which was significantly higher in comparison with healthy controls (19%). In addition, EBV expression gradually increased from 27% (CIN1) to 35% (CIN2/3). EBV coinfection with HPV also posed a fourfold increased risk of cervical cancer in EBV-positive women (12); similarly, precancerous cervical lesions were twice as common in EBV-positive women compared with EBV-negative cases (12). Taken together, these data indicate EBV as a potentially active cofactor (not only passenger/bystander) in the cervical cancer pathogenesis and progression.

Most cervical carcinomas are invasive squamous cell carcinomas (keratinizing and non-keratinizing types). However, many other subtypes have been recognized including lymphoepithelioma-like carcinoma (LELC) of the cervix (40). LELC is a poorly differentiated (non-keratinizing) cervical carcinoma with rich inflammatory stroma, composed predominantly of T-lymphocytes (CD4+ and CD8+) with minor component of B-lymphocytes (CD20+ and CD79a+) and NK-cells (CD56+) (41–43). It is a distinct variant of cervical carcinoma that may also be associated with HPV infection (44) although some studies revealed no HPV infection in LELC (43, 45). Morphologically, it is similar to its nasopharyngeal counterpart that is a prototype of cancer associated with EBV infection (46). LELC exhibits some unique clinicopathologic characteristics including affection of younger patients, presentation at earlier stage, and more favorable outcome compared with conventional cervical carcinoma (45, 47). Although some studies reported association of LELC with EBV infection in Asian women (47, 48), other studies failed to confirm this observation including other ethnic groups (e.g., Caucasians) (44, 49–54). In addition, the study of Chao et al. using real-time PCR and EBV-encoded RNA *in situ* hybridization revealed the EBV sequences in a florid inflammatory stromal component, but not in poorly differentiated squamous cells of LELC (45). In contrast, EBV presence has been documented in neoplastic cells of the lymphoepithelial carcinomas at other locations (e.g., salivary and lacrimal glands, middle ear, larynx, pancreas, and esophagus) (55–60).

In addition, EBV infection has been demonstrated in several lymphoproliferative lesions of the cervix, e.g., lymphoma-like lesions (35, 61, 62) and extranodal NK/T-cell lymphoma (63).

### HPV and EBV in Cervical Cancer

One of the most intriguing research issues is the possible synergistic effects of HPVs and EBV in promoting cervical carcinogenesis and progression. Such synergism and coinfections have already been observed in nasopharyngeal carcinoma, particularly the variant from the endemic regions (China and Southeast Asia) (14) and oral squamous cell carcinoma (11, 64).

**Figure 2:**
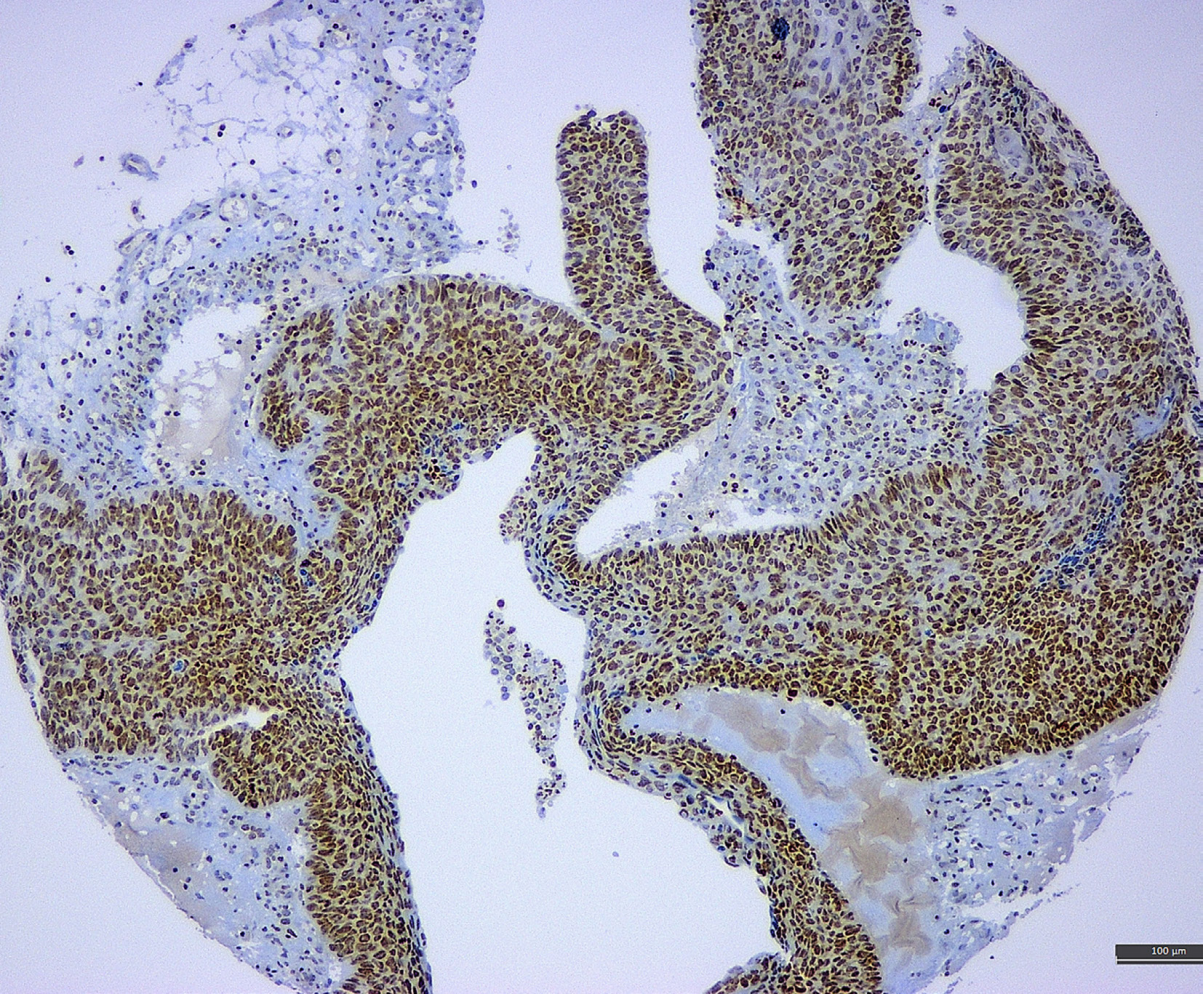
A tissue microarray sample of cervical cancer associated with CIN3 lesion exhibiting strong positivity for Epstein–Barr virus (latent membrane protein 1 antibody) (immunohistochemistry, 10×).

A recent meta-analysis of de Lima et al. (12) revealed a HPV/EBV coinfection rate in cervical carcinoma to be ~29%. They also found a positive association between EBV load and lesion grade (from CIN1 to CIN3 and invasive cancer), indicating a potential causal role of EBV in cervical carcinogenesis and progression (an illustration of EBV positivity in CIN3 lesion and cervical carcinoma is shown in Figure 2). One of the possible scenarios is a transformation of cervical cells *via* C3d complement receptor that is widely expressed in cervix, making cervical cells more sensitive to various oncogenic stimuli (8). EBV presence in cervix may also accelerate integration of HPV genome into cervical cell’s genome, enhancing genomic instability of the infected cervical cells (65). In addition, chronic cervicitis, a common condition during female reproductive life, may also facilitate the EBV infection and its potential oncogenic effects (8). We have also proposed that viruses alone or in collaboration may induce oncogene activation and promote the epithelial–mesenchymal transition, one of the key steps in cancer progression and metastasis (Figure 1) (66).

As correctly observed in the meta-analysis of de Lima et al. (12), EBV along with cytomegalovirus (CMV) may also be detected in cervical secretions and uterine cervix from healthy young women. Several studies confirmed the frequency of EBV and CMV in cervical secretions among healthy women to be between 10 and 30% (67–70). Such findings may also be clinically relevant given that viruses such as CMV, HSVs, and varicella zoster may cause congenital, perinatal, or neonatal infections (67). The role of vertical transmission of EBV is still uncertain although rare cases of congenital EBV infection have been reported (71, 72).

The discrepant data on EBV positivity rate in cervical may be caused by the different diagnostic assays, the sample type, and other technical issues that may affect the results (e.g., automated vs. manual detecting system) (73). Thus, PCR is a highly sensitive method, but it cannot discriminate between neoplastic (epithelial) and stromal/inflammatory cells (e.g., B-lymphocytes) giving the false-positive results (74) while *in situ* hybridization and immunohistochemical assays (e.g., ISH RNA and specific antibodies against EBV antigens, see Figure 2) may be more helpful to precisely identify the viral load in the specific cell compartments. Of note, most studies exploring and reporting EBV positivity in cervical carcinoma used only PCR-based assays (65, 75–78). One study reported EBV positivity by PCR in CIN3 lesions and cervical carcinoma in 15 and 5.8%, respectively, while ISH RNA (EBER) revealed no EBV positivity (0%) in any of the tested samples (79). This led the authors to conclude that EBV plays little role in the pathogenesis of cervical carcinoma in their population (79). Similarly, another study reported EBV positivity in 40/58% (69%) of cervical carcinoma samples by PCR while immunohistochemistry (LMP1 protein) revealed EBV positivity in only 26% of tested samples (*n* = 23) (80). In contrast, Szkaradkiewicz et al. reported a significantly higher detection rate of EBV positivity among CIN3 lesions by ISH (70%) in comparison with PCR-based assay (30%), but the sample size was small (*n* = 10) (68). Of note, many of the reported studies employed small number of cases (e.g., McCormick used 18 invasive carcinoma samples while Aromseree et al. used only four invasive carcinoma samples for ISH assay). On the other hand, commercially available immunohistochemical assays (e.g., antibodies against EBNA1 or LMP1 proteins) may be affected by the antibody specificity and sensitivity and preanalytical issues (tissue fixation and processing). In all detection assays, differences in sample preparation (cytology: cell block vs. cytospin, biopsy: small vs. surgical; frozen tissue vs. formalin-fixed tissue) and sampling technique (swab, spatula, or cytobrush) may also have a significant impact on the obtained results and may account for the reported discrepancies (12, 76, 81).

### Interplay Between PD-L1 and EBV in Cervical Carcinoma and Other EBV-Related Malignancies

Recent breakthrough advances in cancer treatment are mainly due to the therapeutic effects of immune checkpoint inhibitors [such as anti-programmed cell death-1 (PD-1)/PD-L1] that have revolutionized management of several cancers including non-small-cell lung carcinoma, renal cell carcinoma, advanced urothelial carcinoma, Merkel cell carcinoma, microsatellite instable (MSI-H) colorectal carcinoma, malignant melanoma, and classical HL (82–84). The interaction between PD-1 and its ligand PD-L1 enables cancer cells to escape T-cell-mediated cellular cytotoxicity by suppressing the function of T-lymphocytes. Numerous studies have described the mechanisms of PD-L1 activation in tumor cells (85). One of the mechanisms of PD-L1 upregulation may be *via* EBV in EBV-associated malignancies such as gastric carcinoma and classical HL (86, 87). Thus, in the case of EBV+ gastric carcinomas, EBNA1 may induce both constitutive and IFNγ-inducible PD-L1 expression in EBV (+) gastric carcinoma cells (Figure 1) (86). Compared with EBV (−) gastric cells, EBV (+) gastric cells showed significantly higher PD-L1 expression by activating the JAK2/STAT1/IRF-1 signaling pathway (86). On the other hand, activation of inhibitory PD-1/PD-L1 axis may allow for immune evasion of EBV-associated cancer cells (Figure 1) (88). In contrast, two separate studies on nasopharyngeal carcinoma revealed tumor cells’ PD-L1 expression in 44 and 64% of the cases, respectively, but PD-L1 status was not associated with EBV viral load (89, 90).

PD-L1 expression has been described in substantial proportion of cervical carcinomas (both squamous cell and adenocarcinomas) and precursor (CIN) lesions of the cervix (91–93). A study of Yang-Chun et al. (93) demonstrated a positive association between HPV and PD-L1 status in CIN lesions and invasive cervical carcinoma. The status of PD-L1 in LELC of cervix is unknown as no studies are available at present. The data on pulmonary LELC indicate PD-L1 expression in cancer cells and a favorable therapeutic response to a PD-1 inhibitor nivolumab (94, 95). Though by no means conclusive, the little available data presented in these studies provide speculative fuel to the notion that there might be an important interplay between immune checkpoint proteins and EBV in cervical and possibly other cancers. However, further studies are needed to precisely identify any interplay between EBV and PD-L1 in CIN lesions and cervical carcinomas.

## Conclusion and Future Directions

Epstein–Barr virus infections play a prominent role in cancer initiation and progression in several human malignancies including several lymphomas (both B- and T-cell lineages) and carcinomas (nasopharyngeal and gastric). Current evidence suggests a possible causal relationship between EBV and cervical cancer pathogenesis. A commonly present coinfection of EBV and HPV in cervical cancer (such as oral cancer) also indicates a potential oncogenic interplay between the two viruses. More studies (both basic/experimental and clinical/observational with larger sample size) are necessary to elucidate the oncogenic relevance of the copresence and its clinical impact. The lack of basic studies on PD-L1 and EBV interplay in cervix also merits further research. Given the success of cervical cancer prevention through HPV vaccination and upcoming EBV vaccine, additional molecular and translational/clinical studies on EBV are necessary to allow for the further improvements in its prevention, particularly in developing countries that are affected by the highest rates of infections (HPV and EBV) and cervical cancer burden.

## Author Contributions

SV and A-EM conceived the review. SV searched the literature. SV, FC, SA, and A-EM critically appraised the literature, wrote, and approved final version of the manuscript.

## Conflict of Interest Statement

The authors declare that the research was conducted in the absence of any commercial or financial relationships that could be construed as a potential conflict of interest.

## References

[1] Vedham V Verma M Mahabir S. Early-life exposures to infectious agents and later cancer development. Cancer Med (2015) 4:1908–22. 10.1002/cam4.538 26377256 PMC4940808

[2] Mui UN Haley CT Tyring SK. Viral oncology: molecular biology and pathogenesis. J Clin Med (2017) 6:E111. 10.3390/jcm6120111 29186062 PMC5742800

[3] Martin D Gutkind JS. Human tumor-associated viruses and new insights into the molecular mechanisms of cancer. Oncogene (2008) 27(Suppl 2):S31–42. 10.1038/onc.2009.351 19956178

[4] Weiss RA Tumour-inducing viruses. Br J Hosp Med (Lond) (2016) 77:565–8. 10.12968/hmed.2016.77.10.565 27723397

[5] Parkin DM. The global health burden of infection-associated cancers in the year 2002. Int J Cancer (2006) 118:3030–44. 10.1002/ijc.21731 16404738

[6] Esau D Viral causes of lymphoma: the history of Epstein-Barr virus and human T-lymphotropic virus 1. Virology (Auckl) (2017) 8 10.1177/1178122X17731772 PMC5621661 28983187

[7] De Flora S La Maestra S. Epidemiology of cancers of infectious origin and prevention strategies. J Prev Med Hyg (2015) 56:E15–20. 26789827 PMC4718340

[8] Shi Y Peng SL Yang LF Chen X Tao YG Cao Y. Co-infection of Epstein-Barr virus and human papillomavirus in human tumorigenesis. Chin J Cancer (2016) 35:16. 10.1186/s40880-016-0079-1 26801987 PMC4724123

[9] Antonsson A Wilson LF Kendall BJ Bain CJ Whiteman DC Neale RE. Cancers in Australia in 2010 attributable to infectious agents. Aust N Z J Public Health (2015) 39:446–51. 10.1111/1753-6405.12445 26437730 PMC4606775

[10] zur Hausen H. Papillomaviruses in the causation of human cancers – a brief historical account. Virology (2009) 384:260–5. 10.1016/j.virol.2008.11.046 19135222

[11] Al Moustafa A-E Cyprian FS Al-Antary N Yasmeen A High-Risk Human Papillomaviruses and Epstein-Barr Virus Presence and Crosstalk in Human Oral Carcinogenesis. Cham: Springer International Publishing AG (2017).

[12] de Lima MAP Neto PJN Lima LPM Goncalves Junior J Teixeira Junior AG Teodoro IPP Association between Epstein-Barr virus (EBV) and cervical carcinoma: a meta-analysis. Gynecol Oncol (2017) 148(2):317–28. 10.1016/j.ygyno.2017.10.005 29021084

[13] Vockerodt M Yap LF Shannon-Lowe C Curley H Wei W Vrzalikova K The Epstein-Barr virus and the pathogenesis of lymphoma. J Pathol (2015) 235:312–22. 10.1002/path.4459 25294567

[14] Guidry JT Scott RS. The interaction between human papillomavirus and other viruses. Virus Res (2017) 231:139–47. 10.1016/j.virusres.2016.11.002 27826043 PMC5325789

[15] Ghosh SK Perrine SP Faller DV. Advances in virus-directed therapeutics against Epstein-Barr virus-associated malignancies. Adv Virol (2012) 2012:509296. 10.1155/2012/509296 22500168 PMC3303631

[16] Nakanishi Y Wakisaka N Kondo S Endo K Sugimoto H Hatano M Progression of understanding for the role of Epstein-Barr virus and management of nasopharyngeal carcinoma. Cancer Metastasis Rev (2017) 36:435–47. 10.1007/s10555-017-9693-x 28819752 PMC5613035

[17] Chen L Qiu X Zhang N Wang Y Wang M Li D APOBEC-mediated genomic alterations link immunity and viral infection during human papillomavirus-driven cervical carcinogenesis. Biosci Trends (2017) 11:383–8. 10.5582/bst.2017.01103 28717061

[18] Kalchschmidt JS Bashford-Rogers R Paschos K Gillman AC Styles CT Kellam P Epstein-Barr virus nuclear protein EBNA3C directly induces expression of AID and somatic mutations in B cells. J Exp Med (2016) 213:921–8. 10.1084/jem.20160120 27217538 PMC4886369

[19] Vieira VC Soares MA. The role of cytidine deaminases on innate immune responses against human viral infections. Biomed Res Int (2013) 2013:683095. 10.1155/2013/683095 23865062 PMC3707226

[20] She Y Nong X Zhang M Wang M. Epstein-Barr virus infection and oral squamous cell carcinoma risk: a meta-analysis. PLoS One (2017) 12:e0186860. 10.1371/journal.pone.0186860 29065191 PMC5655447

[21] Whitaker NJ Glenn WK Sahrudin A Orde MM Delprado W Lawson JS. Human papillomavirus and Epstein Barr virus in prostate cancer: koilocytes indicate potential oncogenic influences of human papillomavirus in prostate cancer. Prostate (2013) 73:236–41. 10.1002/pros.22562 22851253

[22] Al Moustafa AE Al-Antary N Aboulkassim T Akil N Batist G Yasmeen A. Co-prevalence of Epstein-Barr virus and high-risk human papillomaviruses in Syrian women with breast cancer. Hum Vaccin Immunother (2016) 12:1936–9. 10.1080/21645515.2016.1139255 27082145 PMC4964818

[23] Mozaffari HR Ramezani M Janbakhsh A Sadeghi M. Malignant salivary gland tumors and Epstein-Barr virus (EBV) infection: a systematic review and meta-analysis. Asian Pac J Cancer Prev (2017) 18:1201–6. 10.22034/APJCP.2017.18.5.1201 28610402 PMC5555523

[24] Cohen JI. Epstein-Barr virus vaccines. Clin Transl Immunol (2015) 4:e32. 10.1038/cti.2014.27 25671130 PMC4318489

[25] Rajcani J Banati F Szenthe K Szathmary S. The potential of currently unavailable herpes virus vaccines. Expert Rev Vaccines (2018) 17:239–48. 10.1080/14760584.2018.1425620 29313728

[26] Sokal EM Hoppenbrouwers K Vandermeulen C Moutschen M Leonard P Moreels A Recombinant gp350 vaccine for infectious mononucleosis: a phase 2, randomized, double-blind, placebo-controlled trial to evaluate the safety, immunogenicity, and efficacy of an Epstein-Barr virus vaccine in healthy young adults. J Infect Dis (2007) 196:1749–53. 10.1086/523813 18190254

[27] Small W Jr Bacon MA Bajaj A Chuang LT Fisher BJ Harkenrider MM Cervical cancer: a global health crisis. Cancer (2017) 123:2404–12. 10.1002/cncr.30667 28464289

[28] Torre LA Bray F Siegel RL Ferlay J Lortet-Tieulent J Jemal A. Global cancer statistics, 2012. CA Cancer J Clin (2015) 65:87–108. 10.3322/caac.21262 25651787

[29] Echelman D Feldman S. Management of cervical precancers: a global perspective. Hematol Oncol Clin North Am (2012) 26:31–44. 10.1016/j.hoc.2011.11.005 22244660

[30] Munoz N Bosch FX De Sanjose S Herrero R Castellsague X Shah KV Epidemiologic classification of human papillomavirus types associated with cervical cancer. N Engl J Med (2003) 348:518–27. 10.1056/NEJMoa021641 12571259

[31] Sixbey JW Lemon SM Pagano JS. A second site for Epstein-Barr virus shedding: the uterine cervix. Lancet (1986) 2:1122–4. 10.1016/S0140-6736(86)90531-3 2877273

[32] Landers RJ O’Leary JJ Crowley M Healy I Annis P Burke L Epstein-Barr virus in normal, pre-malignant, and malignant lesions of the uterine cervix. J Clin Pathol (1993) 46:931–5. 10.1136/jcp.46.10.931 8227411 PMC501621

[33] Sasagawa T Shimakage M Nakamura M Sakaike J Ishikawa H Inoue M. Epstein-Barr virus (EBV) genes expression in cervical intraepithelial neoplasia and invasive cervical cancer: a comparative study with human papillomavirus (HPV) infection. Hum Pathol (2000) 31:318–26. 10.1016/S0046-8177(00)80245-2 10746674

[34] Wong KY Collins RJ Srivastava G Pittaluga S Cheung AN Wong LC. Epstein Barr virus in carcinoma of the cervix. Int J Gynecol Pathol (1993) 12:224–7. 10.1097/00004347-199307000-00004 8393845

[35] Hachisuga T Ookuma Y Fukuda K Iwasaka T Sugimori H Watanabe T. Detection of Epstein-Barr virus DNA from a lymphoma-like lesion of the uterine cervix. Gynecol Oncol (1992) 46:69–73. 10.1016/0090-8258(92)90199-S 1321783

[36] Hilton DA Brown LJ Pringle JH Nandha H. Absence of Epstein-Barr virus in carcinoma of the cervix. Cancer (1993) 72:1946–8. 10.1002/1097-0142(19930915)72:6<1946::AID-CNCR2820720625>3.0.CO;2-7 8395968

[37] Payne S Kernohan NM Walker F. Absence of in situ hybridization evidence for latent- or lytic-phase Epstein-Barr virus infection of preinvasive squamous lesions of the cervix. J Pathol (1995) 176:221–6. 10.1002/path.1711760303 7674084

[38] O’Leary JJ Landers RJ Crowley M Healy I Kealy WF Hogan J Genotypic mapping of HPV and assessment of EBV prevalence in endocervical lesions. J Clin Pathol (1997) 50:904–10. 10.1136/jcp.50.11.904 9462238 PMC500313

[39] Shoji Y Saegusa M Takano Y Hashimura M Okayasu I. Detection of the Epstein-Barr virus genome in cervical neoplasia is closely related to the degree of infiltrating lymphoid cells: a polymerase chain reaction and in situ hybridization approach. Pathol Int (1997) 47:507–11. 10.1111/j.1440-1827.1997.tb04532.x 9293529

[40] Kurman RJ Carcangiu ML Herrington CS Young RH WHO Classification of Tumours of Female Reproductive Organs. IARC (2014).

[41] Mills SE Austin MB Randall ME. Lymphoepithelioma-like carcinoma of the uterine cervix. A distinctive, undifferentiated carcinoma with inflammatory stroma. Am J Surg Pathol (1985) 9:883–9. 10.1097/00000478-198512000-00004 3934992

[42] Philippe A Rassy M Craciun L Naveaux C Willard-Gallo K Larsimont D Inflammatory stroma of lymphoepithelioma-like carcinoma of the cervix: immunohistochemical study of 3 cases and review of the literature. Int J Gynecol Pathol (2017). 10.1097/PGP.0000000000000446 28985196

[43] Takai N Nakamura S Goto K Hayashita C Kira N Urabe S Lymphoepithelioma-like carcinoma of the uterine cervix. Arch Gynecol Obstet (2009) 280:725–7. 10.1007/s00404-009-0993-4 19238413

[44] Noel J Lespagnard L Fayt I Verhest A Dargent J. Evidence of human papilloma virus infection but lack of Epstein-Barr virus in lymphoepithelioma-like carcinoma of uterine cervix: report of two cases and review of the literature. Hum Pathol (2001) 32:135–8. 10.1053/hupa.2001.20901 11172309

[45] Chao A Tsai CN Hsueh S Lee LY Chen TC Huang SL Does Epstein-Barr virus play a role in lymphoepithelioma-like carcinoma of the uterine cervix? Int J Gynecol Pathol (2009) 28:279–85. 10.1097/PGP.0b013e31818fb0a9 19620947

[46] Young LS Dawson CW. Epstein-Barr virus and nasopharyngeal carcinoma. Chin J Cancer (2014) 33:581–90. 10.5732/cjc.014.10197 25418193 PMC4308653

[47] Tseng CJ Pao CC Tseng LH Chang CT Lai CH Soong YK Lymphoepithelioma-like carcinoma of the uterine cervix: association with Epstein-Barr virus and human papillomavirus. Cancer (1997) 80:91–7. 10.1002/(SICI)1097-0142(19970701)80:1<91::AID-CNCR12>3.0.CO;2-A 9210713

[48] Tseng LH Tseng CJ Soong YK Hsueh S Pao CC. Evidence of Epstein-Barr virus in lymphoepithelioma-like carcinoma of the uterine cervix: a case report. Changgeng Yi Xue Za Zhi (1998) 21:184–8. 9729653

[49] Bais AG Kooi S Teune TM Ewing PC Ansink AC. Lymphoepithelioma-like carcinoma of the uterine cervix: absence of Epstein-Barr virus, but presence of a multiple human papillomavirus infection. Gynecol Oncol (2005) 97:716–8. 10.1016/j.ygyno.2005.01.016 15863191

[50] Kohrenhagen N Eck M Holler S Dietl J. Lymphoepithelioma-like carcinoma of the uterine cervix: absence of Epstein-Barr virus and high-risk human papilloma virus infection. Arch Gynecol Obstet (2008) 277:175–8. 10.1007/s00404-007-0427-0 17674013

[51] Takebayashi K Nishida M Matsumoto H Nasu K Narahara H. A case of lymphoepithelioma-like carcinoma in the uterine cervix. Rare Tumors (2015) 7:5688. 10.4081/rt.2015.5688 25918614 PMC4387360

[52] Lopez-Rios F Miguel PS Bellas C Ballestin C Hernandez L. Lymphoepithelioma-like carcinoma of the uterine cervix: a case report studied by in situ hybridization and polymerase chain reaction for Epstein-Barr virus. Arch Pathol Lab Med (2000) 124:746–7. 10.1043/0003-9985(2000)124<0746:LLCOTU>2.0.CO;2 10782160

[53] Matias-Guiu X Komminoth P Prat J Absence of Epstein-Barr virus DNA in lymphoepithelioma-like carcinoma of the the uterine cervix. Am J Clin Pathol (1994) 101:117 10.1093/ajcp/101.1.117 8279448

[54] Weinberg E Hoisington S Eastman AY Rice DK Malfetano J Ross JS. Uterine cervical lymphoepithelial-like carcinoma. Absence of Epstein-Barr virus genomes. Am J Clin Pathol (1993) 99:195–9. 10.1093/ajcp/99.2.195 8382447

[55] Maeda H Yamashiro T Yamashita Y Hirakawa H Agena S Uehara T Lymphoepithelial carcinoma in parotid gland related to EBV infection: a case report. Auris Nasus Larynx (2017) 45(1):170–4. 10.1016/j.anl.2016.12.010 28139343

[56] Kermani W Belcadhi M Sriha B Abdelkefi M. Epstein-Barr virus-associated lymphoepithelial carcinoma of the larynx. Eur Ann Otorhinolaryngol Head Neck Dis (2015) 132:231–3. 10.1016/j.anorl.2015.05.004 26043818

[57] Samdani RT Hechtman JF O’Reilly E Dematteo R Sigel CS. EBV-associated lymphoepithelioma-like carcinoma of the pancreas: case report with targeted sequencing analysis. Pancreatology (2015) 15:302–4. 10.1016/j.pan.2015.03.016 25922198 PMC5029421

[58] Keelawat S Tirakunwichcha S Saonanon P Maneesri-Legrand S Ruangvejvorachai P Lerdlum S Cytokeratin-negative undifferentiated (lymphoepithelial) carcinoma of the lacrimal sac. Ophthal Plast Reconstr Surg (2017) 33:e16–8. 10.1097/IOP.0000000000000422 25719371

[59] Terada T. Epstein-Barr virus associated lymphoepithelial carcinoma of the esophagus. Int J Clin Exp Med (2013) 6:219–26. 23573354 PMC3609699

[60] Huon LK Wang PC Huang SH Epstein Barr virus-associated lymphoepithelial carcinoma in the middle ear. Otolaryngol Head Neck Surg (2011) 144:296–7. 10.1177/0194599810390479 21493437

[61] Okamura I Ikeda T Epstein-Barr virus associated with a lymphoma-mimicking lesion of the uterine cervix. Blood (2016) 128:1439 10.1182/blood-2016-06-722520 27769038

[62] Ramalingam P Zoroquiain P Valbuena JR Kemp BL Medeiros LJ. Florid reactive lymphoid hyperplasia (lymphoma-like lesion) of the uterine cervix. Ann Diagn Pathol (2012) 16:21–8. 10.1016/j.anndiagpath.2011.08.004 22056039

[63] Omori M Oishi N Nakazawa T Nakazawa K Mitsumori T Yuminamochi T Extranodal NK/T-cell lymphoma, nasal type of the uterine cervix: a case report. Diagn Cytopathol (2016) 44:430–3. 10.1002/dc.23439 26872300

[64] Al Moustafa AE Chen D Ghabreau L Akil N. Association between human papillomavirus and Epstein-Barr virus infections in human oral carcinogenesis. Med Hypotheses (2009) 73:184–6. 10.1016/j.mehy.2009.02.025 19361933

[65] Kahla S Oueslati S Achour M Kochbati L Chanoufi MB Maalej M Correlation between EBV co-infection and HPV16 genome integrity in Tunisian cervical cancer patients. Braz J Microbiol (2012) 43:744–53. 10.1590/S1517-83822012000200039 24031886 PMC3768824

[66] Al Moustafa AE Achkhar A Yasmeen A EGF-receptor signaling and epithelial-mesenchymal transition in human carcinomas. Front Biosci (Schol Ed) (2012) 4:671–84. 10.2741/s292 22202084

[67] Berntsson M Dubicanac L Tunback P Ellstrom A Lowhagen GB Bergstrom T. Frequent detection of cytomegalovirus and Epstein-Barr virus in cervical secretions from healthy young women. Acta Obstet Gynecol Scand (2013) 92:706–10. 10.1111/aogs.12134 23550605

[68] Szkaradkiewicz A Wal M Kuch A Pieta P. Human papillomavirus (HPV) and Epstein-Barr virus (EBV) cervical infections in women with normal and abnormal cytology. Pol J Microbiol (2004) 53:95–9. 15478354

[69] Enbom M Strand A Falk KI Linde A Detection of Epstein-Barr virus, but not human herpesvirus 8, DNA in cervical secretions from Swedish women by real-time polymerase chain reaction. Sex Transm Dis (2001) 28:300–6. 10.1097/00007435-200105000-00013 11354271

[70] Staykova J Belovska T Murad A Kakid S Nacheva A Shikova E. Cervical viral infections among asymptomatic Bulgarian women. Cent Eur J Public Health (2016) 24:176–9. 10.21101/cejph.a4299 27760284

[71] Schuster V Janssen W Seidenspinner S Kreth HW [Congenital Epstein-Barr virus infection]. Monatsschr Kinderheilkd (1993) 141:401–4. 8392141

[72] Gumbo H Chasekwa B Church JA Ntozini R Mutasa K Humphrey JH Congenital and postnatal CMV and EBV acquisition in HIV-infected Zimbabwean infants. PLoS One (2014) 9:e114870. 10.1371/journal.pone.0114870 25522217 PMC4270791

[73] Fanaian NK Cohen C Waldrop S Wang J Shehata BM. Epstein-Barr virus (EBV)-encoded RNA: automated in-situ hybridization (ISH) compared with manual ISH and immunohistochemistry for detection of EBV in pediatric lymphoproliferative disorders. Pediatr Dev Pathol (2009) 12:195–9. 10.2350/07-07-0316.1 18442302

[74] Korabecna M Ludvikova M Skalova A. Molecular diagnosis of Epstein-Barr virus in paraffin-embedded tissues of tumors with abundant lymphoid infiltration. Neoplasma (2003) 50:8–12. 12687272

[75] McCormick TM Canedo NH Furtado YL Silveira FA De Lima RJ Rosman AD Association between human papillomavirus and Epstein-Barr virus DNA and gene promoter methylation of RB1 and CDH1 in the cervical lesions: a transversal study. Diagn Pathol (2015) 10:59. 10.1186/s13000-015-0283-3 26032781 PMC4450846

[76] Aromseree S Pientong C Swangphon P Chaiwongkot A Patarapadungkit N Kleebkaow P Possible contributing role of Epstein-Barr virus (EBV) as a cofactor in human papillomavirus (HPV)-associated cervical carcinogenesis. J Clin Virol (2015) 73:70–6. 10.1016/j.jcv.2015.10.015 26551071

[77] Silver MI Paul P Sowjanya P Ramakrishna G Vedantham H Kalpana B Shedding of Epstein-Barr virus and cytomegalovirus from the genital tract of women in a periurban community in Andhra Pradesh, India. J Clin Microbiol (2011) 49:2435–9. 10.1128/JCM.02206-10 21525227 PMC3147891

[78] Santos NB Villanova FE Andrade PM Ribalta J Focchi J Otsuka AY Epstein-Barr virus detection in invasive and pre-invasive lesions of the uterine cervix. Oncol Rep (2009) 21:403–5. 10.3892/or_00000236 19148514

[79] Seo SS Kim WH Song YS Kim SH Kim JW Park NH Epstein-Barr virus plays little role in cervical carcinogenesis in Korean women. Int J Gynecol Cancer (2005) 15:312–8. 10.1111/j.1525-1438.2005.15222.x 15823118

[80] Khenchouche A Sadouki N Boudriche A Houali K Graba A Ooka T Human papillomavirus and Epstein-Barr virus co-infection in cervical carcinoma in Algerian women. Virol J (2013) 10:340. 10.1186/1743-422X-10-340 24252325 PMC4225508

[81] Garady C Saieg MA Ko HM Geddie WR Boerner SL Da Cunha Santos G. Epstein-Barr virus encoded RNA detected by in situ hybridization using cytological preparations. Cytopathology (2014) 25:101–7. 10.1111/cyt.12073 23725487

[82] Imamovic D Vranic S Novel regulators of PD-L1 expression in cancer: CMTM6 and CMTM4-a new avenue to enhance the therapeutic benefits of immune checkpoint inhibitors. Ann Transl Med (2017) 5:467 10.21037/atm.2017.09.32 29285500 PMC5733317

[83] Postow MA Callahan MK Wolchok JD. Immune checkpoint blockade in cancer therapy. J Clin Oncol (2015) 33:1974–82. 10.1200/JCO.2014.59.4358 25605845 PMC4980573

[84] Emens LA Ascierto PA Darcy PK Demaria S Eggermont AMM Redmond WL Cancer immunotherapy: opportunities and challenges in the rapidly evolving clinical landscape. Eur J Cancer (2017) 81:116–29. 10.1016/j.ejca.2017.01.035 28623775

[85] Zou W Wolchok JD Chen L. PD-L1 (B7-H1) and PD-1 pathway blockade for cancer therapy: mechanisms, response biomarkers, and combinations. Sci Transl Med (2016) 8:328rv4. 10.1126/scitranslmed.aad7118 26936508 PMC4859220

[86] Moon JW Kong SK Kim BS Kim HJ Lim H Noh K IFNgamma induces PD-L1 overexpression by JAK2/STAT1/IRF-1 signaling in EBV-positive gastric carcinoma. Sci Rep (2017) 7:17810 10.1038/s41598-017-18132-0 29259270 PMC5736657

[87] Merryman RW Armand P Wright KT Rodig SJ. Checkpoint blockade in Hodgkin and non-Hodgkin lymphoma. Blood Adv (2017) 1:2643–54. 10.1182/bloodadvances.2017012534 29296917 PMC5728646

[88] Goodman A Patel SP Kurzrock R. PD-1-PD-L1 immune-checkpoint blockade in B-cell lymphomas. Nat Rev Clin Oncol (2017) 14:203–20. 10.1038/nrclinonc.2016.168 27805626

[89] Zhou Y Shi D Miao J Wu H Chen J Zhou X PD-L1 predicts poor prognosis for nasopharyngeal carcinoma irrespective of PD-1 and EBV-DNA load. Sci Rep (2017) 7:43627. 10.1038/srep43627 28256540 PMC5335261

[90] Chang AMV Chiosea SI Altman A Pagdanganan HA Ma C. Programmed death-ligand 1 expression, microsatellite instability, Epstein-Barr virus, and human papillomavirus in nasopharyngeal carcinomas of patients from the Philippines. Head Neck Pathol (2017) 11:203–11. 10.1007/s12105-016-0765-y 27807760 PMC5429283

[91] Heeren AM Punt S Bleeker MC Gaarenstroom KN Van Der Velden J Kenter GG Prognostic effect of different PD-L1 expression patterns in squamous cell carcinoma and adenocarcinoma of the cervix. Mod Pathol (2016) 29:753–63. 10.1038/modpathol.2016.64 27056074 PMC4931542

[92] Kim M Kim H Suh DH Kim K Kim H Kim YB Identifying rational candidates for immunotherapy targeting PD-1/PD-L1 in cervical cancer. Anticancer Res (2017) 37:5087–94. 10.21873/anticanres.11926 28870938

[93] Yang-Chun F Zhen-Zhen C Yan-Chun H Xiu-Min M. Association between PD-L1 and HPV status and the prognostic value for HPV treatment in premalignant cervical lesion patients. Medicine (Baltimore) (2017) 96:e7270. 10.1097/MD.0000000000007270 28640134 PMC5484242

[94] Kumar V Dave V Harris J Huang Y. Response of advanced stage recurrent lymphoepithelioma-like carcinoma to nivolumab. Immunotherapy (2017) 9:955–61. 10.2217/imt-2017-0067 28971752

[95] Jiang L Wang L Li PF Zhang XK Chen JW Qiu HJ Positive expression of programmed death ligand-1 correlates with superior outcomes and might be a therapeutic target in primary pulmonary lymphoepithelioma-like carcinoma. Onco Targets Ther (2015) 8:1451–7. 10.2147/OTT.S84234 26109869 PMC4474388

